# In-Situ Synthesis, Microstructure, and Mechanical Properties of TiB_2_-Reinforced Fe-Cr-Mn-Al Steel Matrix Composites Prepared by Spark Plasma Sintering

**DOI:** 10.3390/ma14092346

**Published:** 2021-04-30

**Authors:** Jian Liu, Min Wu, Jian Chen, Zibo Ye, Cheng Lin, Weiping Chen, Canyi Du

**Affiliations:** 1 School of Automobile and Transportation Engineering, Guangdong Polytechnic Normal University, Guangzhou 510665, China; aloysliu@gpnu.edu.cn (J.L.); yezibo@gpnu.edu.cn (Z.Y.); lincheng@gpnu.edu.cn (C.L.); 2 School of Mechatronic Engineering, Guangdong Polytechnic Normal University, Guangzhou 510665, China; jianchen@gpnu.edu.cn; 3 Guangdong Key Laboratory for Advanced Metallic Materials Processing, South China University of Technology, Guangzhou 510640, China; mewpchen@scut.edu.cn

**Keywords:** in-situ synthesis, Fe-Cr-Mn-Al, TiB_2_, composites, spark plasma sintering

## Abstract

In-situ synthesis, microstructure, and mechanical properties of four TiB_2_-Reinforced Fe-Cr-Mn-Al Steel Matrix Composites have been researched in this work. The microstructure and phases of the prepared specimens have been characterized by using scanning electron microscopy (SEM), X-ray diffraction technique, and transmission electron microscopy (TEM). The sintered specimens consisted of Fe_2_AlCr, CrFeB-type boride, and TiB_2_. The mechanical properties, such as hardness and compression strength at room temperature (RT) and at elevated temperatures (600 °C and 800 °C) have been evaluated. The compressive strength and Vickers hardness of the sintered specimens increase with the volume fraction of TiB_2_ in the matrix, which are all much higher than those of the ex-situ TiB_2_/Fe-15Cr-20Mn-8Al composites and the reported TiB_2_/Fe-Cr composites with the same volume fraction of TiB_2_. The highest Vickers hardness and compressive strength at room temperature are 1213 ± 35 HV and 3500 ± 20 MPa, respectively. As the testing temperature increases to 600 °C, or even 800 °C, these composites still show relatively high compressive strength. Precipitation strengthening of CrFeB and in-situ synthesis of TiB_2_ as well as nanocrystalline microstructure produced by the combination of mechanical alloying (MA) and spark plasma sintering (SPS) can account for the high Vickers hardness and compressive strength.

## 1. Introduction

The ever-growing demand for lightweight materials represents one main challenge for structural materials design in current transportation systems and machine parts. High-strength structural steel is one of the lightweight selections for automobiles. These steels with high-strength-to-weight ratio are provided as thinner gauge sheet steel in order to decrease the weight of car and modify crash worthiness [1,2]. It is well known that the reduction of vehicle weight is mainly achieved by reducing the thickness of steel plate when high strength steel is used. However, reducing the thickness of the material to a certain extent will meet the bottleneck of stiffness. Therefore, directly reducing the material density is another way to further reduce the weight of components effectively, based on high strength.

At present, Fe-Mn-Al-C steels with lower density than the traditional high strength steels are getting great interest in potential applications for automotive structural parts because of their special feature of increase of the tensile or yield strength and the ductility at the same time. However, high amounts of Al and Mn will reduce these steels’ Young modulus and further deteriorate the component stiffness, which are the main problems that prevent applying Fe-Mn-Al-C steel in automotive lightweight fields.

It is well known that Metal Matrix Composites (MMCs) are being used increasingly in the automotive industry [3], cutting tools [4,5,6], and aerospace [7], due to their strength, quality, and light weight. Ceramic particle reinforced metal matrix composites are composed of ductile metal matrix and ceramic particle reinforced matrix, which have good plastic toughness and high strength stiffness. Desired enhancement of elastic modulus and decrease of thickness could be accomplished by introducing of appropriate ceramic particles into steels. Since TiB_2_ with high melting temperature (2980 °C) has high modulus of elasticity, high hardness, good chemical inertness, and corrosion resistance, it is the best reinforced selection for steel matrix, compared with various ceramic particulates. Guo [8] studied iron-based composites reinforced with different volume fraction of 15%, 20%, and 25% of TiB_2_ obtained by in-situ synthesis method and showed that the modulus and hardness of Fe-TiB_2_ increased with TiB_2_ content. Kulikowski et al. [9] showed that additions of TiB_2_ resulted in reduced density, increased stiffness and specific stiffness in the composite compared with the matrix when studying mechanical properties of TiB_2_ reinforced Fe and 316 L stainless steel composites. However, the addition of such inherently brittle ceramic particles significantly deteriorates the materials’ toughness and ductility [10,11]. To optimize the particle’s size, morphology, and dispersion combined with the matrix microstructure modifications by adapted alloying additions is one typical strategy. Among the many elements that are currently available, Mn is considered to be most appropriate to adjust the matrix’ microstructure and improve the co-deformation procedures between particle and matrix, and has only small positive effects on the TiB_2_ microstructure [12]. It has been demonstrated that Al can enhance the stacking fault energy of austenite and facilitate the austenite to decompose into ferrite structure [13], which will affect the mechanical properties. Young pointed that the addition of Cr enhanced the densification of TiB_2_, resulted in more narrow grain size distribution of TiB_2_ and suppressed the coarsening of TiB_2_ grains [14], which is beneficial to the mechanical properties. Therefore, TiB_2_/Fe-Mn-Al-Cr(-C) composite is expected to be a new generation of automotive lightweight composites with high specific strength and high specific stiffness.

Our previous studies showed that TiB_2_/Fe-Cr-20Mn-8Al composites fabricated by ex-situ synthesis from TiB_2_, Cr, Fe, Mn, and Al by MA and SPS consisted of Fe_2_AlCr, CrFeB, Mn_2_B, and TiB_2_, and demonstrated high compressive strength and good hardness [15]. However, it is well known that the properties of composites are related to the matrix constitutions, which are not only related to the chemical composition but also related to the preparation process. Studies [16,17] indicated that different Mn and Al contents resulted in different matrix constitutions and mechanical properties. In addition to this, the ceramic metal interface is an important factor which influences the composite’s structure and properties. Lately, in-situ technique has been applied to synthesize metal matrices with ceramic particulates. In-situ process is advantageous because the chemical reaction to form the dispersed ceramic phase occurs between elements of their compounds, resulting that the new-formed particles are located in the metal matrix and the interfaces have higher interfacial strength, better improved wettability, and more excellent particle-size distribution due to its clean, non-oxidized particle-matrix. Therefore, Mn and Al contents and processing route different from our previous studies are used in this work, aiming to fabricate TiB_2_-reinforced Fe-Cr-Mn-Al matrix composite with better mechanical properties.

## 2. Materials and Methods

### 2.1. Material Preparation

Commercial Cr, Fe, Mn, Al, Ti (the purity is 99.9% in weight and particles size is below 45 μm), and B (the purity is 99.9% in weight and particles size is below 5 μm) were exactly weighed and physically mixed according to the designed nominal compositions of 15, 10, 25, and 30 vol.%TiB_2_-reinforced Fe-15Cr-10Mn-5Al steel matrix composites using a Turbula Mixer (Zibo Qixing New Material Corp. Ltd, Zibo, China). 5 h of mixing later, 100 g mixed elemental powders were mechanically milled in a ball mill with high energy planetary (QM-3SP4) (Nanjing NanDa Instrument Corp, Nanjing, China). The milling of powders was carried out at 300 rpm for 60 h with 304 stainless-steel vials and balls with three diameters of 6 mm, 10 mm, and 20 mm. The ratio of ball to powder was set to 10:1, by weight. After being purged several times, the vials were fed with pure argon gas under 0.4 MPa. The process control agent (PCA) [18] used in this work was Cyclohexane. After being milled for 60 h, the powders were dried and place in a cylindrical die made from graphite whose inside diameter is 20.2 mm, then synthesized by Dr. Sinter 825 SPS. The experimental samples were firstly heated at the rate of 100 °C per min to 600 °C, and then to 1000 °C, while at the rate of 50 °C per min from 1000 °C to 1100 °C. The sintering pressure is 50 MPa, while the holding time is 10 min. During the SPS process, the residual cell pressure of oven chamber is below 8 Pa.

### 2.2. Microstructure and Mechanical Properties Analysis

In order to research the powder phase composition for various milling times and as-sintered bulk specimens, X-ray diffraction patterns were recorded by an X-ray diffractometer (Bruker D8) (Bruker Corp, Billerica, MA, USA) with a 0.1542 nm source (Cu Kα). In order to determine the microstructure of the powders for various milling times, back scattered electron images (BSE) were obtained by using the Phenom proX SEM (scanning electron microscopy) (Phenom world Corp, Eindhoven, Netherlands). In order to determine the microstructure and distribution of the specimens sintered, SEM images and maps were accomplished by using a Zeiss Sigma 500 (Carl Zeiss, Oberkochen, Germany). In addition, the bright field images and SAED (corresponding selected area electron diffraction) patterns were achieved by using a TECNAI G2 S-TWIN F20 TEM (transmission electron microscopy) (FEI, Hillsboro, OR, USA).

The dimension of compression test specimen is Ø4 mm × 6 mm. According to [19], compression tests at room temperature were accomplished by an INSTRON 5569 testing system, while compression tests at elevated temperature were accomplished by a GLEEBLE 3800 testing machine (DSI Corp, Poestenkill, NY, USA). The strain rate used was 1 × 10^−3^ per second. The hardness of the specimens was determined by an HVS-1000 Vickers hardness instrument (Jinan Liling testing machine Corp., Ltd, Jinan, China) with a 300 g load holding about 10 s. A total of 10 locations of each sample were randomly selected to test hardness, and their arithmetic average value was calculated. Finally, each hardness is averaged from samples no less than three.

## 3. Results and Discussion

### 3.1. Powder Characterization

**Figure 1:**
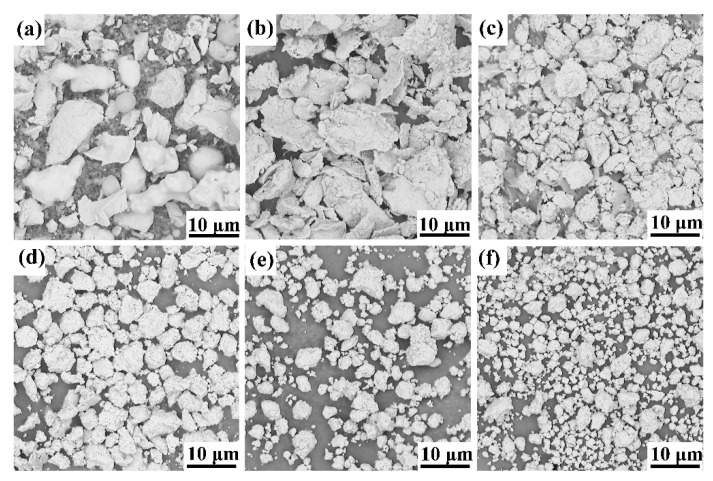
SEM images of 25 vol.%TiB_2_/Fe-15Cr-10Mn-5Al composite powder after (**a**) 0 h, (**b**) 5 h, (**c**) 10 h, (**d**) 20 h, (**e**) 40 h, and (**f**) 60 h of ball milling.

The morphology of the original powders and powder mixture at different stages of high-energy milling of 25 vol.%TiB_2_/Fe-15Cr-10Mn-5Al composite is shown in Figure 1. The 0 h milled powder displays a variety of shapes and sizes in Figure 1a. After milling for 5 h (Figure 1b), rough particles or agglomerates appear, suggesting fracturing and welding for the composite. The critical balance of fracturing and cold welding determines the effect of mechanical alloying [20]. During mechanical alloying, excessive cold welding of soft metallic materials, such as Al, can be restrained by the process control agents used in this work. Further milling to 10 h results in work-hardening, which makes the powder brittle and results in fracture and production of some big particles or agglomerates, as shown in Figure 1c. The shape of these particles becomes irregular again. With continuing milling, the shapes of particles tend to be spherical, and this size range becomes narrow evidently, shown in Figure 1d. After 40 h high-energy milling (Figure 1e), the shape of particles is spherical, and their size tends to decrease to less than 3 μm mostly. Milling for 60 h later, the size distribution is more uniform, and no big agglomerates exists (Figure 1f). Thus, powders become round and uniform, while the average particle size decreases with increasing milling time. Similar results are also observed in mechanical alloying of the other three compositions. The phenomenon mentioned above was also observed in other study [21].

**Figure 2:**
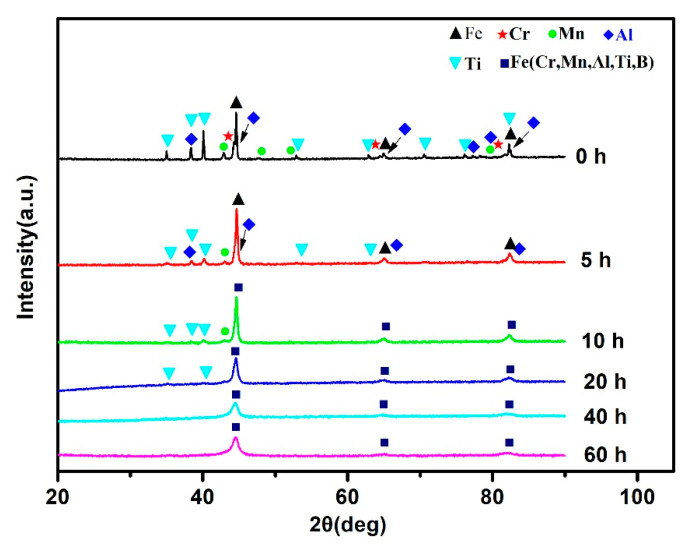
XRD patterns for 25 vol.%TiB_2_/Fe-15Cr-10Mn-5Al powders after different time of ball milling.

Figure 2 illustrates the XRD patterns at different milling times, which is of the mixed and MAed 25 vol.%TiB_2_/Fe-15Cr-10Mn-5Al powders. As for the mixed powder, peaks of Fe, Cr, Mn, Al, and Ti are observed, while the structure of the former three elements is BCC (body cubic centered), Al is FCC, (face cubic centered) and Ti is hexagonal. Fe powders wrap a lot of B powders during blending, which diminishes the content of B outside Fe powders, causing that diffraction peaks of B were very weak and cannot be detected. Peaks of Cr disappear after milling for 5 h, and diffraction peaks corresponding to Mn, Al, and Ti element drastically reduce, indicating that these elements might have entered into Fe lattice with BCC structure little by little, forming the solid solution phase of α-Fe (Cr, Mn, Al, Ti). Based on Chen et al. [22], Al was rapidly disappeared because its melting temperature is low. Nevertheless, Cr atoms and Mn atoms are easy to embed into Fe lattice structure and generate new solid solution since their atomic radius is similar to Fe atom radius, resulting in their early disappearance. Moreover, as the milling time increases, the regions of grain boundary get dominated increasingly, resulting in finely distribution of Cr and Mn on or in the grain boundaries [23]. It is observed that the peak intensity of Al disappears after 10 h milling, and diffraction peaks of Ti further decreases. As the time of milling prolongs to 20 h, the diffraction peak of Mn element cannot be observed and those of Ti element can hardly be found. After 40 h milling, all the elemental diffraction peaks completely disappear. When the time of milling increases to 60 h, there is no evident change of the diffraction peaks. So, we can say that the final product is a metastable BCC Fe-based α-Fe (Cr, Mn, Al, Ti, B) solid solution.

### 3.2. Phase and Microstructure Identification

**Figure 3:**
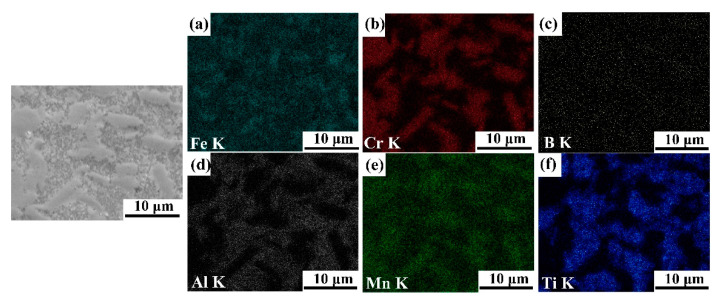
SEM image of 15 vol.%TiB_2_/Fe-15Cr-10Mn-5Al composite and elemental maps showing the distribution of (**a**) Fe, (**b**) Cr, (**c**) B, (**d**) Al, (**e**) Mn, and (**f**) Ti.

**Figure 4:**
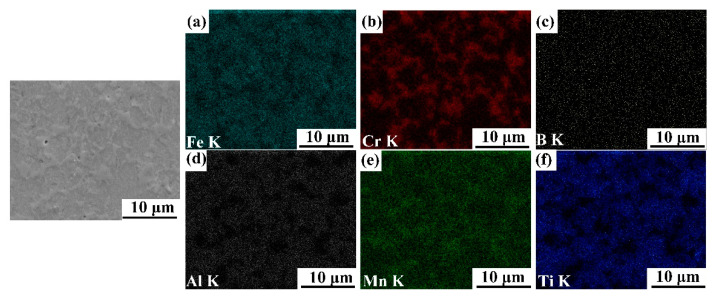
SEM image of 20 vol.%TiB_2_/Fe-15Cr-10Mn-5Al composite and elemental maps showing the distribution of (**a**) Fe, (**b**) Cr, (**c**) B, (**d**) Al, (**e**) Mn, and (**f**) Ti.

**Figure 5:**
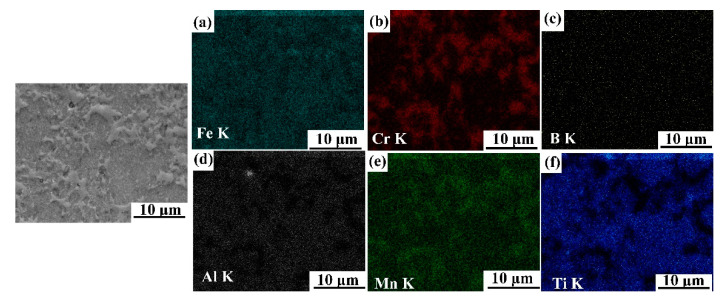
SEM image of 25 vol.%TiB_2_/Fe-15Cr-10Mn-5Al composite and elemental maps showing the distribution of (**a**) Fe, (**b**) Cr, (**c**) B, (**d**) Al, (**e**) Mn, and (**f**) Ti.

**Figure 6:**
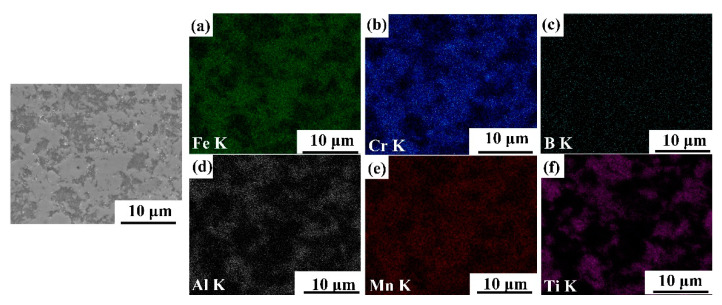
SEM image of 30 vol.%TiB_2_/Fe-15Cr-10Mn-5Al composite and elemental maps showing the distribution of (**a**) Fe, (**b**) Cr, (**c**) B, (**d**) Al, (**e**) Mn, and (**f**) Ti.

Figure 3, Figure 4, Figure 5 and Figure 6 illustrate the SEM images of the four TiB_2_/Fe-15Cr-10Mn-5Al composites sintered and related elemental distribution diagrams. Obviously, four microstructures consist two different phases: Cr-Mn-rich and Al-Ti-lean phase (Figure 3b,e, Figure 4b,e, Figure 5b,e, as well as Figure 6b,e), Ti-Al-rich phase (Figure 3d,f, Figure 4d,f, Figure 5d,f, as well as Figure 6d,f).

**Figure 7:**
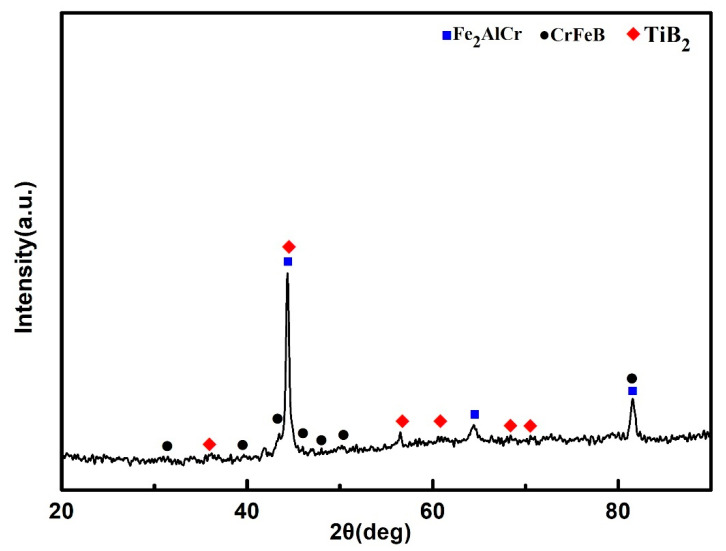
XRD patterns of the 25 vol.%TiB_2_/Fe-15Cr-10Mn-5Al composite sintered at 1100 °C for 10 min under 50 MPa by SPS.

X-ray diffraction pattern for 25 vol.%TiB_2_/Fe-15Cr-10Mn-5Al composite is shown in Figure 7. CrFeB with orthorhombic crystal structure (a is 1.4534 nm, b is 0.7302 nm, and c is 0.4215 nm), Fe_2_AlCr with cubic crystal structure (a is 0.2894 nm), and TiB_2_ with hexagonal crystal structure (a is 0.3036 nm and c is 0.3238 nm) are identified in the composite according to the XRD result. Combined with the SEM result, it is concluded that the Cr-Mn-rich region is complex borides CrFeB, and the Ti-Al-rich region consists of TiB_2_ and Fe_2_AlCr. The main matrix elements are Cr and Fe grouping together in the periodic table, while their properties and parameters of lattice are similar. In addition, Fe_2_AlCr and CrFeB are all intermetallics, which have no precise chemical composition. Hence, to measure the phase composition of these composites precisely just by diffraction analysis with X-ray is quite difficult.

**Figure 8:**
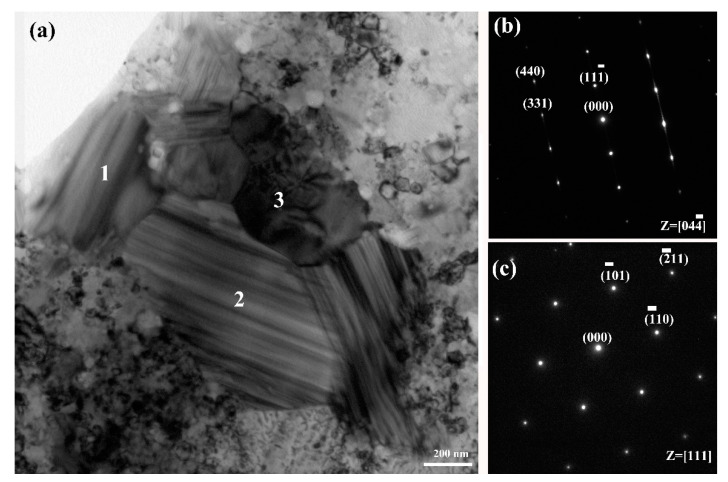
(**a**) TEM bright field image of 25 vol.%TiB_2_/Fe-15Cr-10Mn-5Al composite and three intermetallic grains 1, 2 and 3; (**b**) corresponding SAED demonstrating grain 2 in (**a**) corresponding to CrFeB with orthorhombic crystal symmetry along $\left[04\bar{4}\right]$ zone axis; (**c**) corresponding SAED demonstrating grain 3 in (**a**) corresponding to Fe_2_AlCr with BCC crystal symmetry along [111] zone axis.

Figure 8 illustrates the TEM bright field image 25 vol.%TiB_2_/Fe-15Cr-10Mn-5Al composite and corresponding SAED (selected area electron diffraction) pattern of the two intermetallic phases found in 25 vol.%TiB_2_/Fe-15Cr-10Mn-5Al composite. The related SAED pattern (Figure 8b) suggests that the grain2 has a CrFeB-type structure (a is 1.4534 nm, b is 0.7302 nm, and c is 0.4215 nm) and belongs to Fddd space group. The corresponding SAED pattern (Figure 8c) suggests that the grain 3 has a Fe_2_AlCr-type structure (a is 0.2894 nm) and vests in Im-3 m space group.

Solution-precipitation can account for the generation of TiB_2_, CrFeB, and Fe_2_AlCr in this material. During SPS, some of B powders wrapped by Fe powders during blending react with Ti to form TiB_2_ by solid diffusion process, and the other B elements without enough time to diffuse form Fe_2_B boride by reacting with Fe. Subsequently, part of Fe atoms in the Fe_2_B were replaced by Cr and Mn resulting in forming of CrFeB. Such phenomenon has also been found in other works [24,25,26,27,28]. EDS/TEM as shown in Table 1 indicates that CrFeB is rich in Cr and B, and lack in Al compared with that in the 50 h as-milled powders, which suggests that the neighboring areas around the CrFeB borides would be enriched in Al and their compositions would be turbulent. Therefore, the unstable α phase near to the CrFeB borides changed into the D0_3_-ordered Fe_3_Al in case of 25 vol.%TiB_2_/Fe-15Cr-10Mn-5Al, according to Refs. [29,30]. Part of Fe was replaced subsequently by Cr and Mn, resulting in transformation from phase Fe_3_Al to phase Fe_2_CrAl.

### 3.3. Mechanical Properties

**Figure 9:**
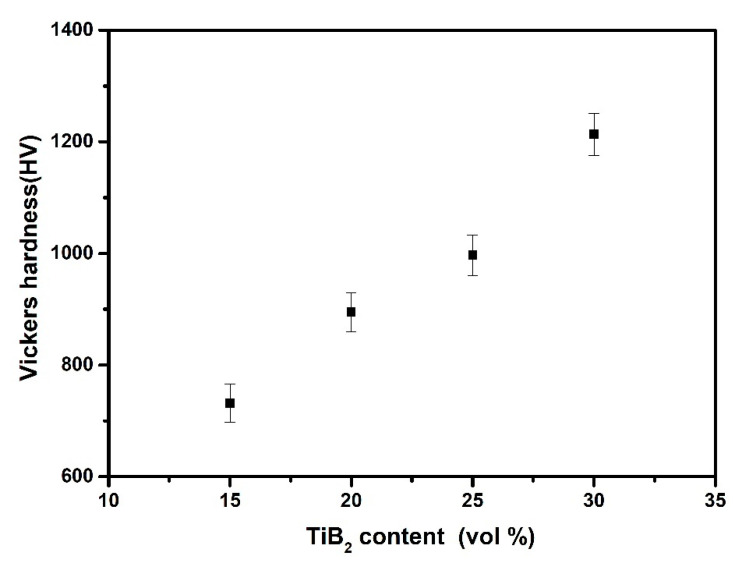
Variation of Vickers hardness of TiB_2_/Fe-15Cr-10Mn-5Al composites with TiB_2_ content.

Figure 9 illustrates the variation of Vickers hardness of TiB_2_/Fe-15Cr-10Mn-5Al composites synthesized at 1100 °C for 10 min as a function of TiB_2_ content. The Vickers hardness were depicted with the arithmetic average of at least three samples and the error bars were depicted by standard deviation. It was observed that TiB_2_ content promotes the increase of Vickers hardness, which might result from the hardness difference between TiB_2_ (25~35 GPa) [31] and CrFeB (22.15 GPa), as well as Fe_2_AlCr (8.04 GPa) [27].

**Figure 10:**
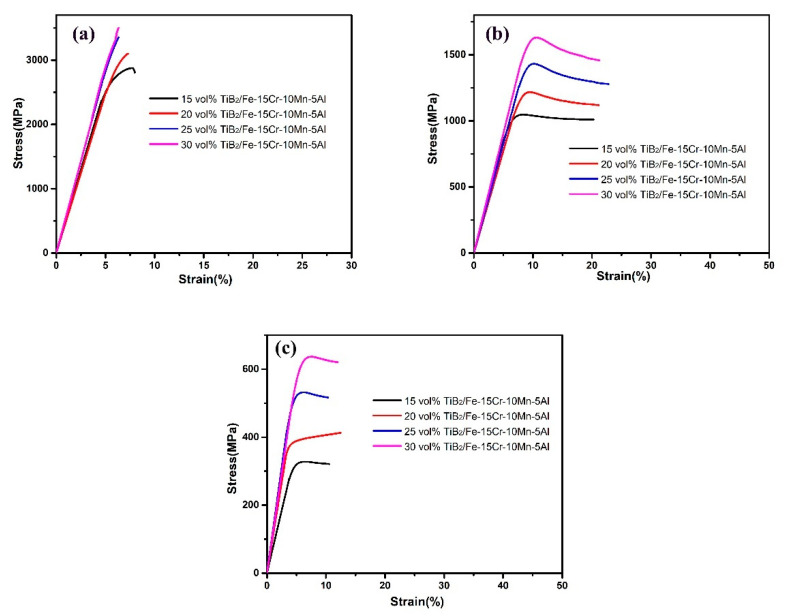
Compressive stress-strain curves obtained for TiB_2_/Fe-15Cr-10Mn-5Al composites with different TiB_2_ at: (**a**) room temperature, (**b**) 600 °C, (**c**) 800 °C.

Figure 10 illustrates the compression testing results of the four SPS sintered TiB_2_/Fe-15Cr-10Mn-5Al composites, which was tested at room and elevated temperatures. In all the composites, high compressive strengths within the ranges of 2877–3500 MPa at ambient temperature (Figure 10a) were gotten. The high compressive strength is obtained by introducing Fe_2_AlCr and CrFeB precipitates with high strength and hardness to the matrix [32], and then by either preventing further grains growth or by refining grains of the matrix or by impeding dislocation migration through TiB_2_ [33]. Additionally, the original compressive stress, which appeared during compression, helps to close the cavity of sintered product, resulting in the delayed initiation of cracks, which lie at the interface of reinforcement and matrix [34]. Detailed results analysis illustrates that the increasing TiB_2_ content of the steel matrix has led the composite compressive strength to increase gradually, which was also observed in test at elevated temperatures, as shown in Figure 10b,c. The reason might be the growing number of interfaces formed in the composite with higher content of TiB_2_ between the composite matrix and the reinforced particles.

**Figure 11:**
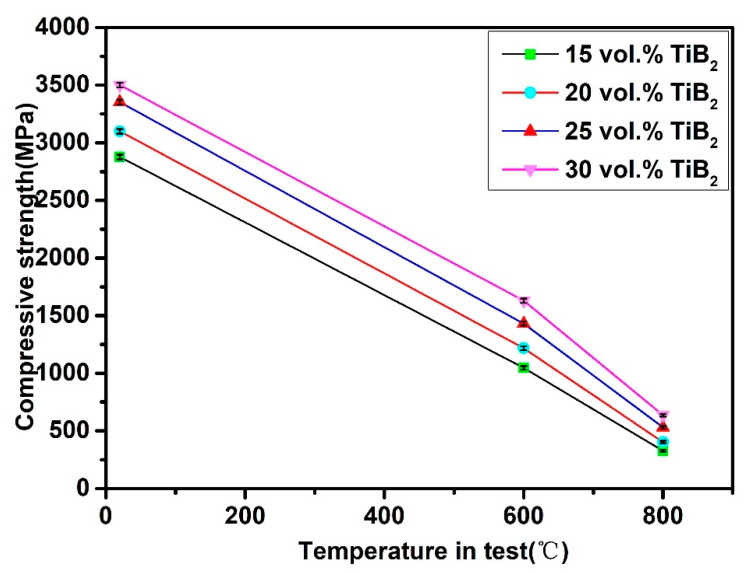
Relationship between test temperature and compressive strength of the sintered TiB_2_/Fe-15Cr-10Mn-5Al composites with different TiB_2_.

Figure 11 illustrates the strength values (in compression tests), which were plotted based on the temperature. Applied temperature has leaded to a gradual reduction of the strengths of the examined samples. The curves had a steep decrease to a level of 327–636 MPa, when the temperature was increased to 800 °C. The SPS sintered steel-TiB_2_ composites had a similar trend [24]. The chief phase of these TiB_2_/Fe-15Cr-10Mn-5Al composites as-sintered by SPS is Fe_2_AlCr with BCC structure, which softens fast at higher temperatures because of the power law creeps after dislocation glide and dislocation climb [35], and thus, the compressive strength at elevated temperatures reduced greatly.

The hardness and compressive strength of the TiB_2_/Fe-15Cr-10Mn-5Al composites researched in this work were compared with the those of ex-situ TiB_2_/Fe-15Cr-20Mn-8Al composite we previously researched [15], and two TiB_2_/ Fe-Cr composites researched by else scholars [36,37], having the same volume fraction of TiB_2_, as shown in Table 2, which were all produced by powder metallurgy. Comparative result shows that TiB_2_/Fe-15Cr-10Mn-5Al composite represents hardness and strength at room temperature much higher than compared with all the other TiB_2_/Fe-Cr composites. On the one hand, there is more hard phases Fe_2_AlCr inTiB_2_/Fe-15Cr-10Mn-5Al composites than in the other TiB_2_/ Fe-Cr composites, as shown in Table 2, which can account for the difference of hardness. On the other hand, according to [38,39], the surface of the reinforcement phases TiB_2_ synthesized directly within the Fe-15Cr-10Mn-5Al matrix by chemical reactions between Ti and B elements during SPS is cleaner and hence the bond between the reinforcing phases and the matrix is stronger compared with the conventional ex-situ methods, which is the other reason for the above-mentioned difference of compressive strength.

## 4. Conclusions

(1) Bulk in-situ TiB_2_ particulate reinforced Fe-15Cr-10Mn-5Al steel matrix composites were successfully synthesized by MA and SPS technique using Fe, Cr, Mn, Al, Ti, and B element powders as original materials.

(2) Phase and microstructure analysis show that the synthesized composites consisted of Fe_2_AlCr, CrFeB-type boride, and TiB_2_.

(3) The hardness and compressive strength of the in-situ TiB_2_/Fe-15Cr-10Mn-5Al composite is higher than those of the ex-situ TiB_2_/Fe-15Cr-20Mn-8Al composite and the reported TiB_2_/Fe-Cr composites with the same volume fraction of TiB_2_.

(4) The hardness and compressive strength of the in-situ TiB_2_/Fe-15Cr-10Mn-5Al composites increase with the TiB_2_ content increasing in the Fe-Cr-Mn-Al matrix from 15 to 30 vol.%, and the highest hardness and compressive strength values equal to 1213 ± 35 HV and 3500 ± 20 MPa.

(5) The compression test temperature has a significant impact on the strength of the sintered composites, resulting in sharply decreasing strength, especially at a temperature of 800 °C.

## Figures and Tables

**Table 1:** Average composition of the phases in 25 vol.%TiB_2_/Fe-15Cr-10Mn-5Al composite (wt.%).

Regions	Phases	Fe	Cr	Mn	Al	B	Ti
Nominal composition	-	57.88	12.40	8.27	4.13	5.40	11.91
1, 2 3	CrFeB Fe_2_AlCr	37.56 79.17	24.64 6.19	9.19 10.48	0 3.91	28.59 0.22	0 0

**Table 2:** The hardness and compressive strength of several TiB_2_/Fe-Cr composites.

Alloys	Process	Hardness (HV)	Compressive Strength (MPa)	Refs
20 vol.%TiB2//Fe-15Cr-10Mn-5Al	MA + SPS	895 ± 35	3100 ± 20 (RT)	This work
20 vol.%TiB2//Fe-15Cr-20Mn-8Al	MA + SPS	670 ± 15	2420 ± 21 (RT)	[15]
20 vol.%TiB2/AISI 316L	HT-HP	460	1350(RT)	[36]
20 vol.%TiB2/AISI 304	HIP	265 *	-	[37]

* Indicates that value was re-estimated according to the corresponding curve [15].

## Data Availability

The data presented in this study are available on request from the corresponding author.
